# FloTrac-guided real-time hemodynamic management for pheochromocytoma resection in a pediatric patient: a case report

**DOI:** 10.3389/fmed.2026.1803223

**Published:** 2026-04-13

**Authors:** Zhao-cheng Zhu, Dao-lin Kang, Wen-fei He, Yan Feng

**Affiliations:** 1 Department of Anesthesiology, Affiliated Hospital of North Sichuan Medical College, Nanchong, China; 2 Department of Pediatric Surgery, Affiliated Hospital of North Sichuan Medical College, Nanchong, China

**Keywords:** anesthetic management, FloTrac, hemodynamics, pediatric anesthesia, pheochromocytoma

## Abstract

Pheochromocytoma remains one of the most challenging endocrine tumors for anesthesiologists, primarily due to catecholamine-induced hemodynamic instability characterized by unpredictable and profound fluctuations. This challenge is further compounded in pediatric patients, whose circulatory reserve, autonomic reactivity, and vascular compliance differ substantially from those of adults. This report details the case of a 14-year-old boy diagnosed with a left adrenal pheochromocytoma who underwent laparoscopic adrenalectomy. Real-time hemodynamic monitoring was performed using the FloTrac/EV1000 platform. In addition to standard parameters, such as arterial blood pressure (ABP) and heart rate (HR), we integrated advanced hemodynamic indices derived from arterial waveform analysis, including cardiac output (CO), cardiac index (CI), stroke volume (SV), stroke volume variation (SVV), pulse pressure variation (PPV), and systemic vascular resistance (SVR). This case report suggested that FloTrac-guided advanced hemodynamic monitoring may facilitate hemodynamic management during pediatric pheochromocytoma surgery.

## Introduction

Pheochromocytoma is an uncommon tumor originating from chromaffin cells of the sympathetic nervous system, characterized by excessive catecholamine secretion (1). The incidence of pheochromocytoma in the pediatric population is low, with an estimated annual rate of 0.3 per million children. The median age at diagnosis typically ranges from 6 to 14 years (2). These tumors lead to the overproduction of catecholamines, including dopamine, epinephrine, and norepinephrine; their pathogenesis may involve genetic or environmental factors (3). Common clinical presentations include headache, hypertension, and tachycardia, with hypertension being particularly prevalent in pediatric cases (4). Consequently, maintaining stringent perioperative hemodynamic control is of paramount importance for patient safety. Although surgical resection represents the definitive curative treatment (5), the procedure itself elevates the risk of hypertensive and hypotensive crises, cardiac arrhythmias, myocardial infarction, and cerebrovascular events due to catecholamine release during tumor manipulation and subsequent withdrawal post-resection (6). The FloTrac system (Edwards Lifesciences, Irvine, CA, United States) is a hemodynamic monitoring platform that leverages arterial waveform analysis to guide real-time intraoperative management. It has emerged as a useful tool for preventing intraoperative hypotension by providing continuous, patient-specific hemodynamic data (7). This system provides key hemodynamic indices, including arterial blood pressure (ABP), cardiac output (CO), cardiac index (CI), stroke volume variation (SVV), pulse pressure variation (PPV), and systemic vascular resistance (SVR) (8). This report describes a case of a 14-year-old boy with a unilateral pheochromocytoma who presented with a brief history of abdominal pain and hypertension. Laboratory tests revealed elevated norepinephrine and normetanephrine levels (with normal epinephrine and metanephrine levels). The patient underwent successful surgical resection under FloTrac-guided hemodynamic monitoring and recovered without complications.

## Case description

**Figure 1:**
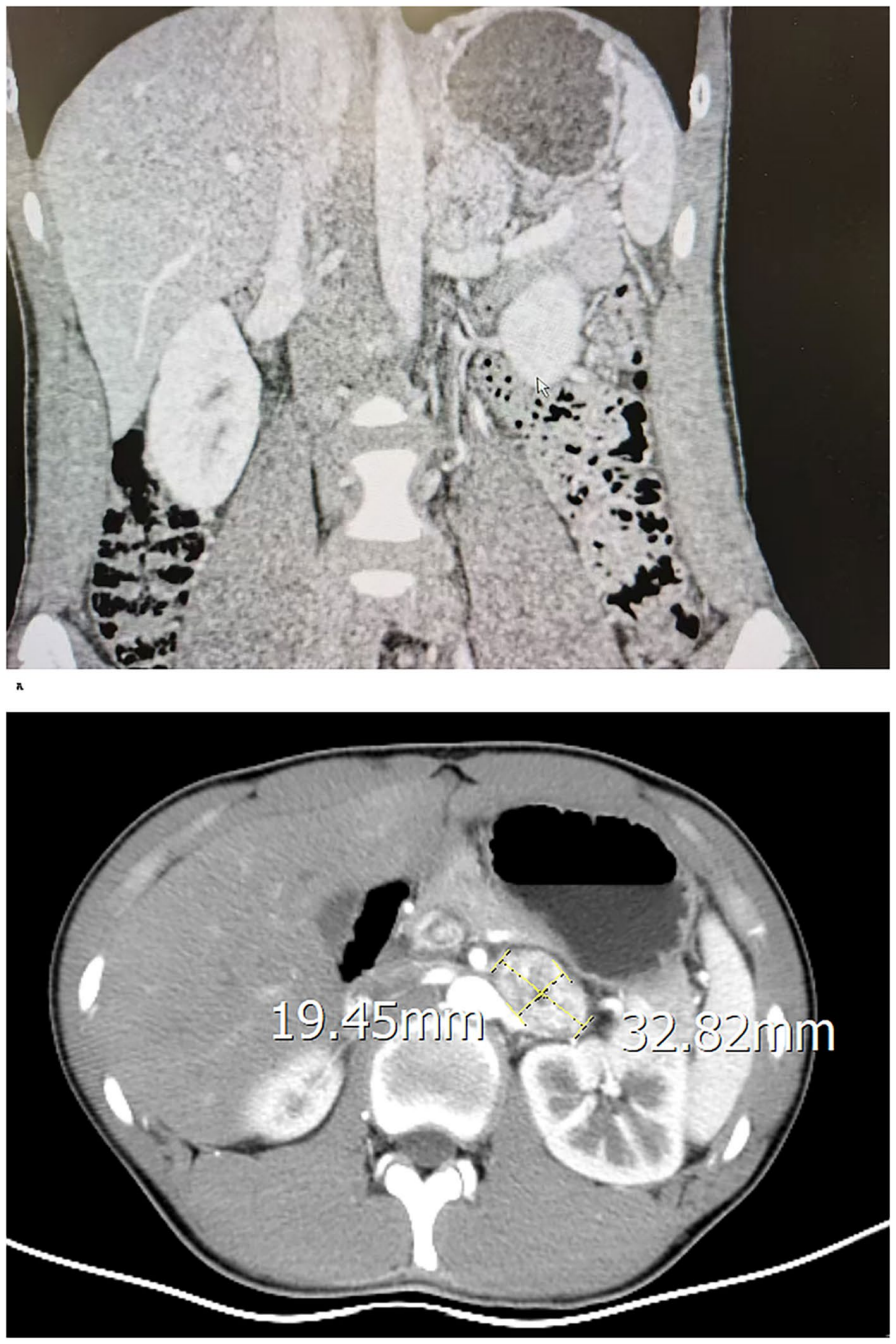
Preoperative abdominal CT showing a 3.3 × 2.0 cm left adrenal pheochromocytoma.

A 14-year-old boy, classified as American Society of Anesthesiologists (ASA) physical status III, was scheduled for elective laparoscopic pheochromocytoma resection. Written informed consent was obtained from his parents before surgery. The patient reported a 4-day history of abdominal pain but denied headache, fever, nausea, vomiting, palpitations, or excessive sweating. Upon admission, physical examination revealed HR of 89 beats per minute, blood pressure of 140/73 mmHg respiratory rate of 20 breaths per minute, temperature of 36.9 °C, which met current pediatric diagnostic criteria for adolescent hypertension (9). No orthostatic blood pressure changes were observed. Cardiorespiratory examination revealed no abnormalities. Abdominal examination revealed a soft, non-tender abdomen without palpable masses, guarding, or organomegaly. Complete blood count, liver and renal function, electrolytes, chest X-ray, and ECG showed no significant abnormalities. Contrast-enhanced abdominal computed tomography demonstrated a well-circumscribed, heterogeneously enhancing left adrenal mass measuring 3.3 × 2.0 cm, with no evidence of local invasion or lymphadenopathy (Figure 1). Plasma norepinephrine and normetanephrine levels were significantly elevated, while epinephrine and metanephrine levels were normal (Table 1). Other endocrine tests were unremarkable, except for a mildly decreased noon ACTH level and an elevated neuron-specific enolase. A diagnosis of left adrenal pheochromocytoma was made.

**Table 1:** Detection of preoperative blood catecholamines and their metabolites.

Item	Method	Result	Flag	Reference range
Norepinephrine	LC-MS/MS	5.22	↑	0.00–5.17 nmol/L
Epinephrine	LC-MS/MS	0.06	—	0.00–0.34 nmol/L
Normetanephrine	LC-MS/MS	12.26	↑	0.00–0.71 nmol/L
Metanephrine	LC-MS/MS	0.12	—	0.00–0.42 nmol/L
Dopamine	LC-MS/MS	<0.05	—	0.00–0.31 nmol/L
3-Methoxytyramine	LC-MS/MS	8.89	—	0.00–18.4 pg./mL
Homovanillic acid	LC-MS/MS	59.70	—	14.27–163.03 nmol/L
Vanillylmandelic acid	LC-MS/MS	60.88	—	0.00–62.00 nmol/L

**Figure 2:**
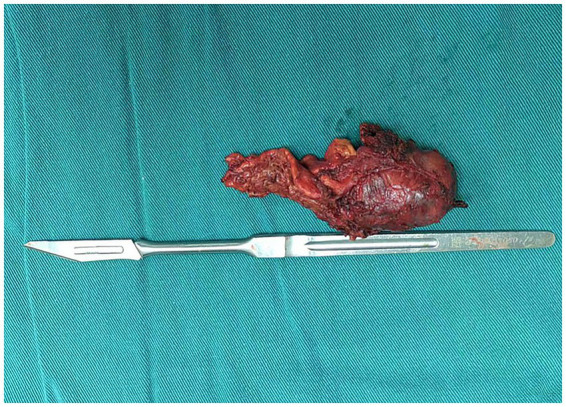
Resected left adrenal tumor specimen.

**Figure 3:**
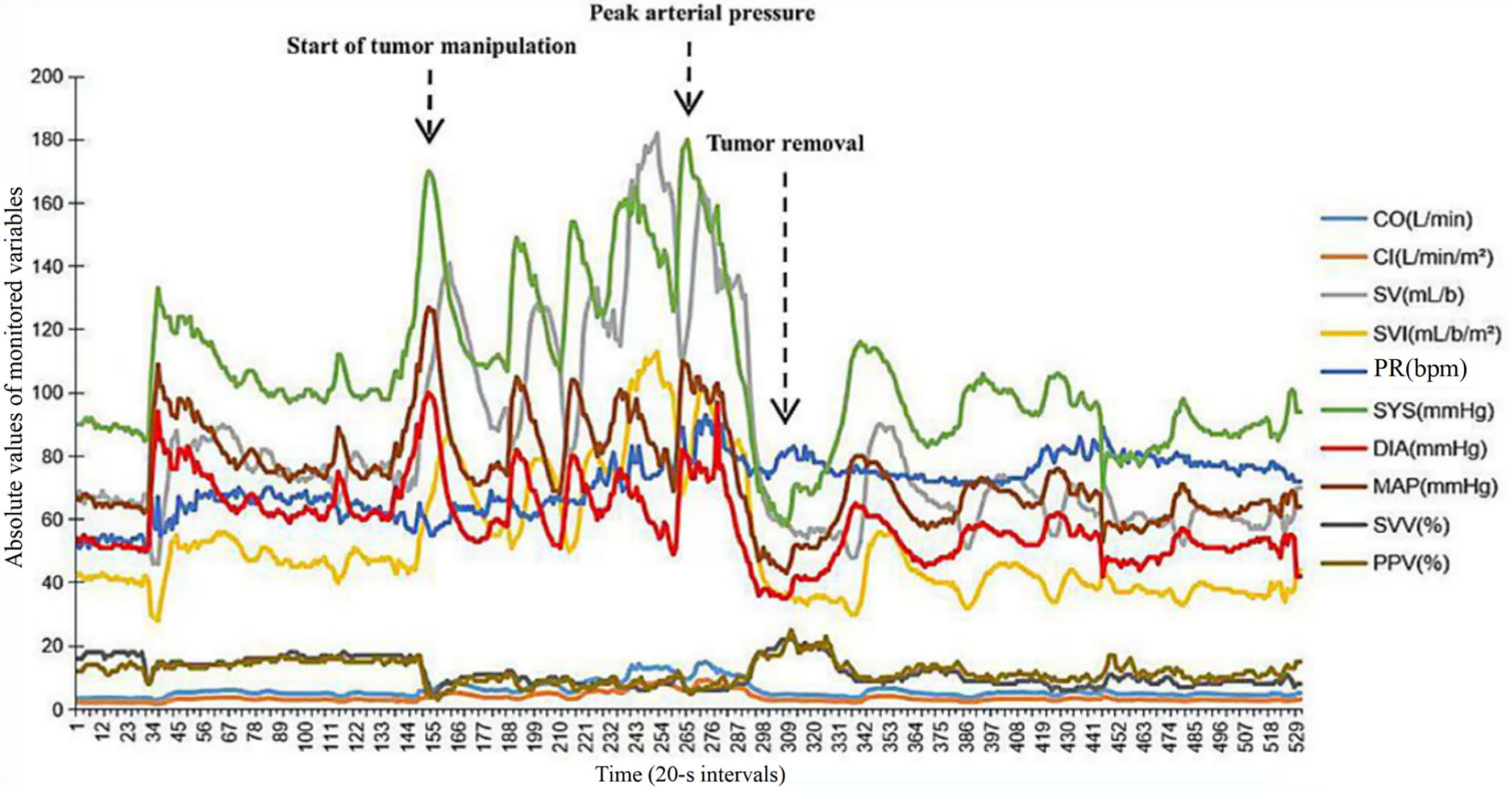
Intraoperative hemodynamic trends recorded by the FloTrac/EV1000 system. Continuous trends of cardiac output (CO), cardiac index (CI), stroke volume (SV), stroke volume index (SVI), pulse rate (PR), systolic arterial pressure (SYS), diastolic arterial pressure (DIA), mean arterial pressure (MAP), stroke volume variation (SVV), and pulse pressure variation (PPV) are displayed from induction of anesthesia to the end of laparoscopic adrenalectomy. The *x*-axis represents time in 20-s intervals, and the *y*-axis represents the absolute values of the monitored variables. Vertical dashed lines indicate the start of tumor manipulation, peak arterial pressure, and tumor removal.

In accordance with established clinical guidelines, the patient received preoperative oral phenoxybenzamine (10 mg three times daily), terazosin, and fluid supplementation. Following the administration of local anesthesia with 1% lidocaine, a left radial arterial catheter was placed. A FloTrac sensor was connected to HemoSphere monitoring platform (Edwards Lifesciences, United States) for continuous assessment of hemodynamic variables (ABP, CO, CI, SVV, PPV, SVR). ABP at pre-induction was 143/79 mmHg. After 5 min of preoxygenation with 100% oxygen, general anesthesia was induced intravenously with propofol (1.5 mg/kg), sufentanil (0.4 μg/kg), cisatracurium (0.15 mg/kg), and remimazolam (0.5 mg/kg). Tracheal intubation and central venous catheter placement were performed. Anesthesia was maintained with 2–3% sevoflurane and continuous infusion of remifentanil (0.1–0.2 μg/kg/min); cisatracurium was administered intermittently to maintain neuromuscular blockade. Prior to surgical incision, the FloTrac monitoring indicated ABP of 107/51 mmHg, CO of 3.5 L/min, SVR of 1,326 dyn·s/cm^5^ and SVV of 10%. During establishment of pneumoperitoneum, ABP remained within the acceptable range. Upon initiation of tumor manipulation, ABP increased abruptly to 170/110 mmHg, accompanied by a rise in SVR to 1,559 dyn·s/cm^5^. We started a continuous intravenous infusion of 3 μg/kg/min of phentolamine, after which ABP and SVR decreased to 110–130/55–70 mmHg and 800–1,000 dyn·s/cm^5^, respectively. During the tumor resection, ABP peaked at 180/80 mmHg while CO was recorded at 14.8 L/min; non-invasive blood pressure (NIBP) was 165/70 mmHg. Whereas SVR showed no significant fluctuation, and a continuous infusion of esmolol at 50 μg/kg/min was followed, hemodynamics were satisfactorily controlled. Once the left adrenal gland vessels were ligated, ABP dropped to 76/47 mmHg, CO decreased to 4.7 L/min and SVV increased to 20%. The esmolol and phentolamine infusions were promptly discontinued. Concurrently, the continuous infusions of norepinephrine at 0.05 μg/kg/min and epinephrine at 0.01 μg/kg/min were initiated, and 500 mL colloidal solutions was administered to reduce the SVV to less than 13%. Hemodynamic parameters were stabilized within minutes, with ABP, CO, SVR and SVV returned to around 110/70 mmHg, 5.5 L/min, 820 dyn·s/cm^5^ and 9%, respectively (Table 2). The norepinephrine and epinephrine infusions were tapered and discontinued prior to wound closure. The patient was extubated 28 min after surgery and transferred to the post-anesthesia care unit for continuous monitoring and recovery. The surgical duration was 167 min. Estimated blood loss was 100 mL, with urine output of 300 mL, 2,100 mL crystalloid solutions and 500 mL colloidal solutions were administrated. Gross complete resection of the tumor was confirmed (Figure 2). Intraoperative hemodynamic trends recorded by the FloTrac system were illustrated in Figure 3. A timeline for hemodynamic parameters and corresponding treatments was shown in Table 2.

**Table 2:** Hemodynamic parameters and interventions.

Item	Pre-incision	Initial tumor manipulation	Peak during manipulation	Immediately after tumor resection	10 min after tumor resection
ABP (mmHg)	107/51	170/110	180/80	76/47	110/70
MAP (mmHg)	67	126	113	55	83
CO (L/min)	3.5	6	14.8	4.7	5.5
SVR (dyn·s/cm^5^)	1,326	1,559	834	447	820
SVV (%)	10	13	6	20	9
Interventions	—	Phentolamine (3 μg/kg/min)	Esmolol (50 μg/kg/min)	Norepinephrine (0.05 μg/kg/min) Epinephrine (0.01 μg/kg/min) Fluid resuscitation	Norepinephrine and epinephrine infusion was tapered off

ABP, arterial blood pressure; MAP, mean arterial pressure; CO, cardiac output; SVV, stroke volume variation; SVV, stroke volume variation.

Postoperative hemodynamics remained stable without notable fluctuations. The subsequent follow-up evaluations, including aldosterone and renin levels, ECG and transthoracic echocardiography were not abnormalities. Pheochromocytoma was confirmed by pathological examination (immunohistochemical staining: CD56+, Syn+, CgA+). The patient was discharged after 1 week.

## Discussion

Pheochromocytoma, rare in children, poses a substantial risk of perioperative hemodynamic instability due to tumor manipulation and the rapid decline in catecholamine concentrations after tumor resection. Extreme fluctuations in ABP, HR, CO, and SVR can precipitate myocardial ischemia, arrhythmias, stroke, or organ hypoperfusion. The present case illustrates the potential utility of an arterial waveform-derived monitoring system (FloTrac) in complementing traditional hemodynamic assessment by providing real-time, flow-based parameters, thereby facilitating precision in physiological management during laparoscopic adrenalectomy in adolescent patients. Previous case reports have largely relied on traditional monitoring, focusing on changes in MAP, HR, and central venous pressure (10, 11). Although this approach reliably detects overt hypertensive or hypotensive crises, it provides limited insight into the underlying hemodynamic mechanisms, specifically in the relative contributions of SVR, SVV, PPV, and CO. This limitation is especially critical in pediatric patients, where age-related variations in vascular compliance, autonomic tone, and circulatory reserve not only complicate hemodynamic interpretation but also predispose to more abrupt and unpredictable responses to catecholamine surges and vasoactive agents compared to adults (12–14).

In adult pheochromocytoma surgery, FloTrac monitoring has proven valuable in characterizing rapid alterations in CO and SVR, facilitating more judicious selection and titration of vasodilators, vasopressors, and intravenous fluids (15). However, its reliability in estimating CO may be limited, especially under conditions of elevated catecholamine levels (16). Accordingly, CO should be interpreted cautiously and conjuncted with SVV and SVR as part of an integrated hemodynamic assessment in pheochromocytoma surgery. In contrast, documented experience with this technology in pediatric settings remains sparse. Previous reports have described the use of FloTrac in pediatric patients, it has been used as an adjunct method rather than the primary hemodynamic monitoring (17). Consequently, this report contributes to the extant evidence by furnishing a comprehensive account of FloTrac-facilitated intraoperative management in an adolescent with a catecholamine-secreting neoplasm. In addition, the FloTrac monitoring system offers distinct advantages over conventional NIBP measurement by providing continuous, real-time hemodynamic data with 20 s interval (18). In the present case, during tumor manipulation, FloTrac recorded an ABP peak of 180/80 mmHg, whereas NIBP peaked at 165/70 mmHg. Following tumor resection, ABP decreased precipitously, yet NIBP readings consistently overestimated the true arterial pressure. Beyond its superior temporal resolution, FloTrac enables precise characterization of the hemodynamic derangements by quantifying key parameters, such as SVV, PPV, CI, CO, and SVR, which collectively inform preload status, myocardial contractility, and vascular tone. This comprehensive, real-time hemodynamic profile empowers clinicians to make timely, targeted decisions and may reduce the incidence of perioperative complications (19).

FloTrac-assisted management not only improves the sensitivity of blood pressure measurement but also effectively prevents paroxysmal hypertension during pheochromocytoma surgery. In adolescent patients who did not receive intraoperative FloTrac-assisted management, blood pressure may rapidly escalate to values such as 223/170 mmHg or 220/130 mmHg, with HR reaching 180 bpm, substantially higher than those observed in patients managed with FloTrac (2, 20). This phenomenon may also be explained by the fact that the patient in the present case exhibited only a modest elevation in preoperative norepinephrine levels without a significant catecholamine release storm. At the start of tumor manipulation, ABP and SVR increased markedly, while CO and SVV remained relatively stable, which suggests that the elevation in ABP was primarily attributable to increased afterload due to vasoconstriction. Therefore, we selected phentolamine to reduce SVR rather than deepening anesthesia or using antihypertensive agents. During the tumor resection, both ABP and CO were increased. As phentolamine was insufficient to control ABP, esmolol was added to regulate the elevated CO, after which hemodynamic parameters were gradually stabilized. Once the left adrenal gland vessels were ligated, profound hypotension was characterized by both the collapse in SVR and the decrease in CO and SVV, indicating that hypotension reflected not only vasodilation due to catecholamine withdrawal, but also relative hypovolemia and long-term catecholamine excess-induced receptor downregulation. Accordingly, a combined therapeutic strategy was instituted, employing norepinephrine infusion to restore vascular tone concurrent with volume resuscitation to optimize preload and CO, rather than treating with vasopressors or fluids in isolation. In this report, the patient was an adolescent with a somatic stature approaching that of adult, the favorable performance of FloTrac in this case may suggest its potential applicability in pediatric patients.

Nevertheless, the present case has limitations and pragmatic consideration. Although FloTrac enhanced our understanding of the underlying hemodynamic mechanisms, our interventions remained predominantly reactive: monitoring was used to interpret established instability rather than to prevent it. The FloTrac platform can be integrated with the hypotension prediction index (HPI), a machine learning based algorithm that analyzes arterial waveform features to predict hypotension before it occurs. Studies in adult patients indicate that HPI-guided management could reduce the duration and severity of intraoperative hypotension and may improve postoperative outcomes (21, 22). However, HPI was not applied in our case. In addition, FloTrac requires an arterial catheter and specific monitoring platform, which may not be available in all pediatric patients, and its accuracy can be affected by extreme vasoconstriction, severe arrhythmias, very low SVR, or altered arterial compliance. The algorithms used by FloTrac have been developed and validated mainly in adults; therefore, their extrapolation to younger or smaller children requires caution and underscores the necessity for dedicated pediatric validation studies. Prospective studies were warranted to determine whether FloTrac combined with HPI translates into measurable improvements in perioperative morbidity and long-term outcomes. Also, our single case report could not demonstrate the superiority of FloTrac-guided management over conventional monitoring.

## Conclusion

This case report demonstrates the value of FloTrac-derived hemodynamic monitoring in guiding perioperative management during laparoscopic adrenalectomy in an adolescent patient. By providing real-time, continuous assessment of flow-based parameters, including CO, SVR, SVV, the FloTrac system enabled precise characterization of the underlying hemodynamic mechanisms driving intraoperative hypertension and hypotension, thereby informing a clinical strategy for the prevention and treatment of pheochromocytoma-related hemodynamic fluctuations. This case adds to the limited evidence supporting advanced hemodynamic monitoring in pediatric pheochromocytoma surgery. Prospective studies were warranted to determine whether FloTrac monitoring could improve perioperative outcomes.

## Data Availability

The original contributions presented in the study are included in the article/supplementary material, further inquiries can be directed to the corresponding author.
